# Parallelization of Curved Inertial Microfluidic Channels to Increase the Throughput of Simultaneous Microparticle Separation and Washing

**DOI:** 10.3390/mi15101228

**Published:** 2024-09-30

**Authors:** Nima Norouzy, Arsalan Nikdoost, Pouya Rezai

**Affiliations:** Department of Mechanical Engineering, York University, BRG 433B, 4700 Keele St., Toronto, ON M3J 1P3, Canada; nimanrzy@yorku.ca (N.N.); nik1991@yorku.ca (A.N.)

**Keywords:** curved microchannel, inertial focusing, particle separation, particle washing, high throughput, parallelization

## Abstract

The rising global need for clean water highlights the importance of efficient sample preparation methods to separate and wash various contaminants such as microparticles. Microfluidic methods for these purposes have emerged but they mostly deliver either separation or washing, with very low throughputs. Here, we investigate parallelization of a curved-channel particle separation and washing device in order to increase its throughput for sample preparation. A curved microchannel applies inertial forces to focus larger 10 µm microparticles at the inner wall of the channel and separate them from smaller 5 µm microparticles at the outer wall. At the same time, Dean flow recirculation is used to exchange the carrier solution of the large microparticles to a clean buffer (washing). We increased the number of curved channels in a stepwise manner from two to four to eight channels in two different arraying designs, i.e., rectangular and polar arrays. We examined efficient separation of target 10 µm particles from 5 µm particles, while transferring the larger microparticles into a clean buffer. Dean flow recirculation studies demonstrated that the rectangular arrayed device performs better, providing solution exchange efficiencies of more than 96% on average as compared to 89% for the polar array device. Our 8-curve rectangular array device provided a particle separation efficiency of 98.93 ± 0.91%, while maintaining a sample purity of 92.83 ± 1.47% at a high working flow rate of 12.8 mL/min. Moreover, the target particles were transferred into a clean buffer with a solution exchange efficiency of 96.81 ± 0.54% in our 8-curve device. Compared to the literature, our in-plane parallelization design of curved microchannels resulted in a 13-fold increase in the working flow rate of the setup while maintaining a very high performance in particle separation and washing. Our microfluidic device offers the potential to enhance the throughput and the separation and washing efficiencies in applications for biological and environmental samples.

## 1. Introduction

The increasing global demand for water has prompted substantial concerns regarding the quality of water resources worldwide. Environmental studies have unveiled the existence of viruses, bacteria, heavy metals, and microparticles such as microplastics (<5 mm in diameter) in natural water sources [1,2]. Separation of target microparticles from other analytes and washing them simultaneously into clean buffers, preferably in a continuous and high throughput manner, can play a pivotal role in on-site water sample preparation and monitoring applications. 

Conventional particle separation methods are predominantly based on label-based isolation [3,4,5,6] or centrifugation. However, these methods are often discontinuous [7] and time-consuming, also requiring skilled labor and bulky and expensive equipment that cannot be easily transported for on-site use at the point of need [8]. Additionally, membrane-based filtration is constrained by membrane pore size and often faces issues with clogging [9]. Microfluidic separation methods on the other hand allow process miniaturization, enabling higher precision, automation, and remote operation [10,11,12].

Microfluidic devices for particle separation are categorized into active and passive methods. Active methods utilize external sources of energy, such as dielectrophoresis [13,14,15], acoustophoresis [16,17,18], or magnetophoresis [19,20,21], to manipulate particles. These methods are complex and limited to low throughputs usually in the range of microliters per minute [14,22,23,24]. In contrast, passive techniques offer a precise control over microparticle migration by manipulation of channel geometry [25] or the flow-induced forces such as inertial and drag forces. The three main types of passive microfluidic techniques include (1) deterministic lateral displacement (DLD) [26,27,28], (2) viscoelastic particle focusing [29,30,31], and (3) inertial microfluidics [32,33].

Inertial forces (Equation (1)) in straight microchannels have been used extensively for microparticles’ manipulation [8,34]. Particle washing in straight microchannels rely on manipulation of particles equilibrium position between two parallel fluid streams using the inertial forces [35,36,37,38,39]. For example, Dudani et al. [38] demonstrated macrovesicles separation and solution exchange in a microfluidic channel at flow rates of 60–70 µL/min. In another report, Sollier et al. [36] utilized an expansion-contraction microchannel to trap large particles, which were subsequently released into a clean buffer using a discontinuous operation process.

(1) $$F_{L}=\rho {\dot{\gamma }}^{2}C_{L}a^{4}$$

Here, the fluid density is denoted by ρ, $\dot{\gamma }$ is the shear rate, *a* is the particle diameter, and *C_L_* stands for the average lift coefficient. The shear rate ($\dot{\gamma }$) can be calculated based on the ratio of maximum axial velocity (*U_max_*) with respect to the channel hydraulic diameter (*D_h_*). While particles move laterally along streamlines within a channel, they face a lateral drag force that acts in the opposite direction of their motion. This force could be approximated by the Stokes drag force [35], when the flow Reynolds number ($Re=\rho UD_{h}/\mu$) is below unity. Here, *U* is the fluid velocity and *µ* represents the fluid dynamic viscosity.

Despite operational simplicities, straight microchannels are usually limited to focusing of particles at multiple points at low flow rates (<100 µL/min) and require a relatively long channel length to facilitate particle focusing [40,41,42,43]. Therefore, simultaneous particle washing and separation cannot be achieved at reasonably high throughputs. 

In curved microchannels [44,45,46,47,48,49,50,51], a secondary flow is created due to the velocity mismatch between the fluid elements along the channel centerline and the ones closer to channel walls. The larger inertia of fluid elements along the channel center pushes them outwards creating a pressure gradient which causes two counter rotating vortices in the lateral direction called Dean vortices [52,53,54]. These vortices are characterized using the non-dimensional Dean number ($De=Re\sqrt{D_{h}/2R}$), where *R* represents the channel radius of curvature. Dean flow exerts a lateral drag force on particles (i.e., Dean drag in Equation (2)) which could alter their equilibrium positions and enable single line focusing in microchannels with a shorter channel footprint [32]. Alternatively, these secondary vortices could be created using expansion-contraction regions [55] and pillared structures inside the microchannels [7].

(2) $$F_{D}=3\pi \mu aV_{De}$$

where *V_De_* represents the average Dean velocity of the secondary vortices [56].

(3) VDe=0.031ϑsDe1.63

Here, ϑ=μρ is the fluid’s kinematic viscosity, and *s* = max (*w*,*h*) is the largest channel dimension, i.e., either *w* or *h* which are the channel width and height, respectively.

A critical issue in utilization of microfluidic devices in industrial and environmental applications is their limited flow rates to process large sample volumes. Various methods have been reported to enhance the operating throughputs in microfluidic devices using channel cross section modifications [57,58,59] and planar parallelization of microchannels [7,60]. Warkiani et al. [61] showcased a multi-layer device with four parallel spiral channels (i.e., a total of 16 spirals) capable of particle focusing with high efficiencies of ~95% with flow rates up to 500 mL/min. They also demonstrated a sized-based separation at a high flow rate of 168 mL/min. Their team also reported a parallelized slanted spiral microchannel for ultra-fast blood plasma separation and achieved a flow rate of 24 mL/min for processing diluted blood samples [62].

In the assessment of biological samples and detection of contamination, a frequently used procedure for sample preparation and post-treatment involves isolating target particles from the initial solution and relocating them to a purified buffer; a process called particle washing in this paper. Considering the shortcomings of straight microchannels, researchers have reported the application of curved microchannels to enhance the efficiencies and working flow rates. Bayat and Rezai [50] introduced a particle washing process using a curved microchannel capable of processing ~10^6^ particles per minute with high separation efficiencies (~86%) and purities (~96%) at a sample flow rate of 1 mL/min. Later on, Fang et al. [63] designed a multilayer centrifuge microfluidic device to extract bacteria and circulating tumor cells (CTCs) using four parallelized spiral channels to separate and enrich target particle concentration. They achieved separation efficiencies of ~85–86% for MCF-7 and A549 cells at flow rates up to 4.0 mL/min.

In spite of recent advancements, there remains a significant gap to enable the simultaneous separation and washing of microparticles at a high throughput and in a continuous manner. Here, we investigate a simple and low-cost curved centrifuge microchannel, and its parallelization in rectangular versus polar arrays, to achieve a high efficiency in particle separation and washing at high flow rates. This design offers potential for further enhancements in throughput using multi-layer stacking, which could prove to be essential in various environmental and industrial applications dealing with large sample volumes.

## 2. Materials and Methods

### 2.1. Sample Preparation

Fluid behavior investigation was performed using undyed and dyed deionized (DI) water, where 15% *v*/*v* trypan blue (Sigma Aldrich, St. Louis, MO, USA) was used to color dye one of the fluid streams inside the microchannels. Two different particle sizes of 4.7 µm (called 5 µm, CM-50-10, 2.5% *w*/*v*), and 10.4 µm (called 10 µm, CM-100-10, 1% *w*/*v*) from Spherotech Inc., Lake Forest, IL, USA were used with approximate concentrations of 10^5^ particles per milliliter to prepare the particles solutions. Tween 20 (Sigma Aldrich, St. Louis, MO, USA) was added to the particles’ solutions with a concentration of 1% *v*/*v* to prevent particle aggregation. Duplex particle separation was performed with a total particle concentration of 10^5^ particles per milliliter.

### 2.2. Microfluidic Device 

Different microfluidic channels were fabricated using standard photolithography and soft lithography techniques. Initially, master molds were prepared by spin coating a layer of SU-8 2075 photoresist (Kayaku Advanced Materials, Newton, MA, USA) on 4-inch silicon wafers (Wafer World Inc., West Palm Beach, FL, USA). This was followed by a prebake process at 65 °C and 95 °C. The wafers were then exposed to UV light (UV-KUB 2, KLEO, Paris, France) with various microchannel photomask designs. A post-bake step at 65 °C and 95 °C was followed by the development process in SU-8 developer solution. Finally, silicon master molds were hard-baked at 200 °C for 20 min.

Microchannels were then fabricated in polydimethylsiloxane (PDMS, Sylgard 18 silicone elastomer kit, Ellsworth Adhesives, Stoney Creek, ON, Canada) using the soft lithography technique [64]. Microchannels were prepared using a 10-to-1 ratio of PDMS prepolymer and the curing agent, which were baked at 75 °C for 3 h. Finally, PDMS microchannels were bonded onto glass slides (Brain Research Laboratories, Newton, MA, USA) using oxygen plasma (Harrick Plasma Inc., Ithaca, NY, USA).

**Figure 1:**
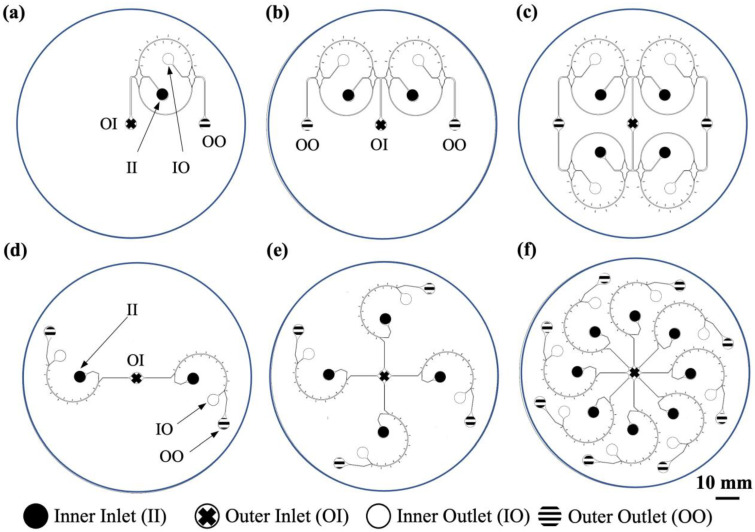
Microchannel arraying designs consisting of 2, 4, and 8 curved channels with *R* = 1.185 cm and *w* × *h* = 300 × 70 µm^2^ cross sections, parallelized in (**a**–**c**) rectangular and (**d**–**f**) polar arrays. Full circles indicate the inner inlets (II), while the outer inlets (OI) are shown with crosses. White circles show the inner outlets (IO), and the outer outlets (OO) are specified with black and white stripes.

Two different channel-arraying configurations were implemented to study the fluid behavior in parallelized devices. As shown in Figure 1, rectangular (Figure 1a–c) and polar (Figure 1d–f) arrays were fabricated with 2, 4, and 8 curve channels. Each curve channel consisted of two inlets to supply the co-flows of dyed and undyed DI water into a 180° curvature with a radius of curvature of *R* = 1.185 cm (*L_curve_* = 3.72 cm), and a cross section of *w* × *h* = 300 × 70 µm^2^. For fluid behavior analysis, the dyed solutions were supplied through the inner inlet (II), alongside a clean buffer at the outer inlet (OI). Particle suspensions were supplied through the inner inlet for the particle behavior investigations. A bifurcated outlet separated the two streams at the end of each curve. These outlets were designed with *w* = 80 µm, and *w* = 160 µm for the inner (IO), and outer outlets (OO), respectively. The inner outlet is designed with a reduced width to collect a purer sample with lower concentrations of carrier fluid. Moreover, this design could enhance the particle separation purity within the inner inlet by confining the focusing area near the inner wall of the channel.

### 2.3. Experimental Procedure

**Figure 2:**
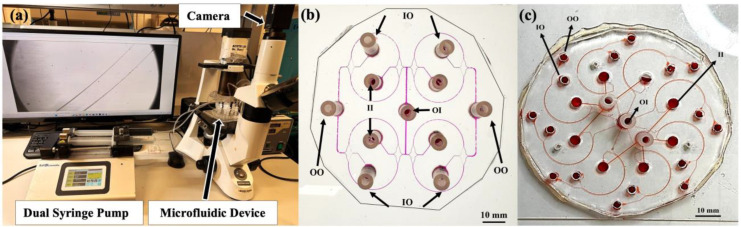
Experimental setup and pictures of 8-curve microfluidic devices. (**a**) Experimental setup, consisting of an inverted microscope equipped wih a camera, a dual syringe pump, and the microfluidic device. (**b**,**c**) PDMS microfluidic devices with 8 curves with a *w* × *h* = 300 × 70 µm^2^ cross section and *R* = 1.185 cm with (**b**) rectangular and (**c**) pollar array designs.

Co-flows of dyed DI water and the clean buffer were run through the curved microchannels at different flow rates using a dual syringe pump (KD Scientific, Holliston, MA, USA) as shown in Figure 2a. An inverted microscope (Bioimager, BIM 500FL, Richmond Hill, ON, Canada) equipped with a camera (FLIR, Richmond, BC, Canada) was used to record the co-flows at 5× magnification. Co-flows in the 2-curve device were supplied at total flow rates of *Q_t_* = 2.4, 3.2, and 4.0 mL/min. These flow rates correspond to *Q_oi_* = *Q_ii_* = 0.6, 0.8, and 1.0 mL/min for each stream of flow in each curve, respectively. Video-recordings were then transferred to the ImageJ software (version 2.9.0) [65,66] for fluid behavior analysis (see next section). 

The fluid exchange purities in 4-curve and 8-curve devices were analyzed using spectrophotometry of the collected samples from the outlets. For particle behavior investigations, particles’ solutions were supplied through the inner inlets (II), alongside a clean DI water buffer. Target particles were then collected at the inner outlet (IO) and analyzed using a hemocytometer (Paul Marienfeld, Lauda-Königshofen, Germany). Each experiment was repeated three times for both fluid and particle behavior. Data analysis steps are thoroughly discussed in the next section.

### 2.4. Data Analysis

#### 2.4.1. Fluid Recirculation Analysis in 2-Curve Devices

Fluid recirculation due to the Dean flow inside the 2-curve designs was analyzed using the obtained videos and the open source software ImageJ [65,66]. Color intensities across lines along the channel width were measured at 10-degree intervals throughout the channel curvature. The standard deviations (*σ*) of these color intensities were used to calculate the switching index, *SI*, with respect to the maximum standard deviation (*σ_max_*), which is observed at the channel inlet, as shown in the Equation (4).

(4) SI=σσmax

**Figure 3:**
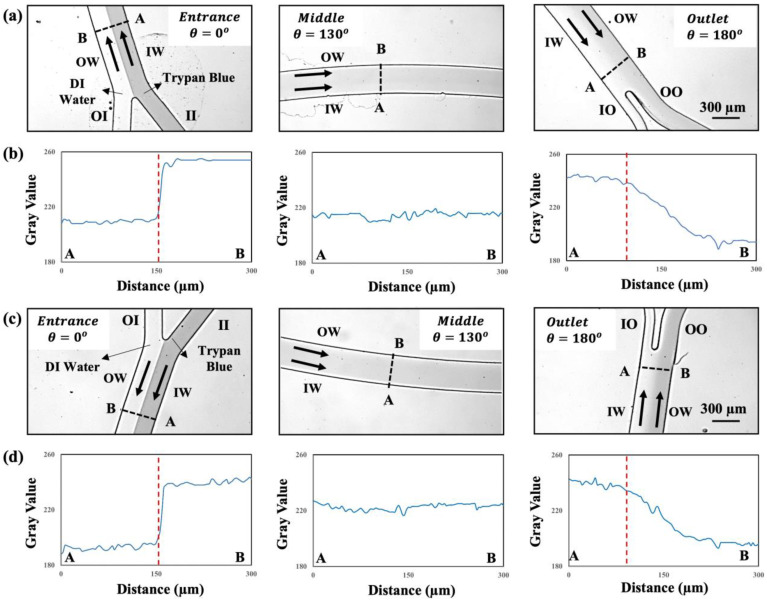
Co-flow images of trypan blue and undyed DI water at a total flow rate of *Q_curve_* = 1.6 mL/min along the microchannel length (*R* = 1.185 cm, *w* × *h* = 300 × 70 µm^2^) in the (**a**) top and (**c**) bottom curves of the 2-curve device with rectangular array. (**b**,**d**) represent the color intensity values along the line A-B in the top and bottom curve of the device, respectively. Red dotted lines indicate the projected locations of the inlet channels (OI and II) or the outlet channels (IO and OO) along line ABs.

Here, the diffusion-based mixing is minimal due to high axial velocity (*V_ax_* ~ 0.95–1.59 m/s) inside the curved channel. Figure 3 shows the channel cross sections at the inlets, middle sections, and the outlets, and the color intensity values alongside the channels’ width for the upper (a–c) and lower (d–f) curves in the rectangular design with two curves, when the liquids are co-flown at a total flow rate of *Q_t_* = 3.2 mL/min (i.e., *Q_curve_* = 1.6 mL/min in each curve). As shown in Figure 3a,c) at the channel inlet, two distinct co-flows of dyed and undyed DI water create the maximum standard deviation (*σ_max_*), which corresponds to the *SI* = 1. At the channel middle section, two co-flow layers have sandwiched each other due to the secondary flow in curvature and create a more uniform color intensity distribution alongside the channel width resulting in a minimum in the standard deviation of color intensity (*σ_min_*), which corresponds to the minimum of switching index (*SI_min_*). Finally, the fluid recirculation at the channel outlet, interchange the location of co-flows, which indicate a 0.5 fluid recirculation or one fluid switch with a local maximum value for the switching index. The required length to reach to the first fluid recirculation is denoted as *L_s_*. As illustrated in Figure 3, the fluid recirculation pattern is similar in both curves in the rectangular curved device, which indicates the uniform performance of our microdevice. In the results section, each experiment was repeated three times and the average *SI* values for each point were drawn against the channel length for the rectangular and polar designs with 2 curves. Standard deviations of these three measurements were reported as the error bars in *SI* graphs.

#### 2.4.2. Fluid Recirculation Analysis in 4- and 8-Curve Devices

Fluid recirculation due to the Dean flow inside 4-curve and 8-curve devices was analyzed using spectrophotometry of the collected samples from the outlets. Solution exchange efficiency was defined as the relative concentration of remainder trypan blue in the collected samples from inner outlets with respect to the initial dyed solution (15% *v*/*v* trypan blue in DI water) at the inner inlet. Here, UV-VIS spectrophotometry (Shimadzu, Kyoto, Japan) was used to measure the absorbance of different trypan blue concentrations in DI water (i.e., 0% *v*/*v* to 2% *v*/*v*). Solution exchange efficiency was then calculated using the Equation (5) below:

(5) $$Solution\;Exchange\;Efficiency=(1-\frac{Trypan\;Blue\;Concentratio\;in\;Inner\;Outlets}{Trypan\;Blue\;Concentration\;in\;Inner\;Inlet})\times 100$$

**Figure 4:**
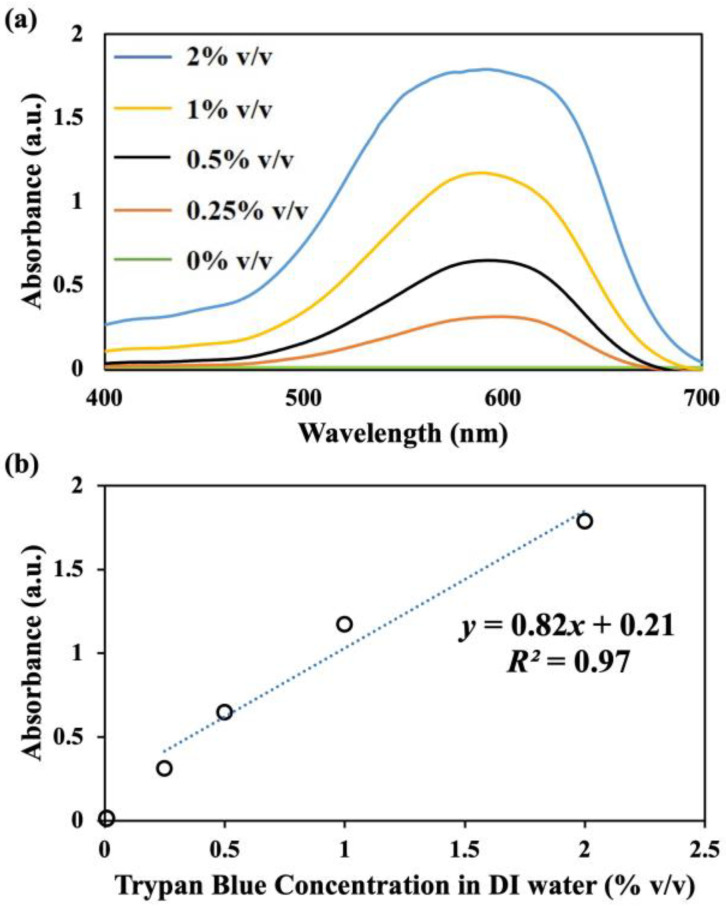
UV-Vis spectrometry of trypan blue spiked water. (**a**) Absorbance of different trypan blue concentrations in DI water in the visible range. Here, 0% *v*/*v* concentration indicates the undyed DI water. (**b**) Absorbance peak at 590 nm vs. trypan blue concentration in DI water. A linear correlation could be fitted over the 0.25–2.0% *v*/*v* range with *R*^2^ = 0.97.

In order to quantify the solution exchange efficiency of the samples collected from channel outlet, we measured the absorbance of various trypan blue concentrations in DI water. As shown in Figure 4a, for a range of 0.25–2.0% *v*/*v*, the peak absorbance in the visible range occurred at an approximate wavelength of ~590 nm. As shown, the peak absorbance increases with the trypan blue concentrations in DI water. These absorbance peaks were then used to fit a linear correlation with respect to the trypan blue concentration in DI water (Figure 4b). 

As shown in Figure 4b, the calibration line in Equation (6) could be used to predict the trypan blue concentration in the collected samples from the outlets with *R*^2^ = 0.97.

(6) y=0.82 x+0.21

Here, *y* stands for the peak absorbance of the solutions and *x* indicates the trypan blue concentration of the collected samples at the channel outlets. The linear correlation in Equation (6) was used to calculate the trypan blue concentrations and the solution exchange efficiencies at the channel outlets.

#### 2.4.3. Particle Separation Efficiency

Particle separation analysis was completed using a hemocytometer (Paul Marienfeld, Lauda-Königshofen, Germany) to count the number of collected particles in each outlet. Collected samples from each outlet were counted manually under the microscope. Particle separation efficiency was defined as shown in Equation (7) below:

(7) $$Efficiency=\frac{Number\;of\;Target\;Particles\;in\;the\;Selected\;Outlet}{Total\;Number\;of\;Target\;Particles\;in\;All\;Outlets}\times 100$$

In the case of duplex particle separation, the purity of the collected samples was calculated using the number of target particles (i.e., 10 µm) in each outlet divided by the total number of collected particles (5 µm and 10 µm) in the respective outlet as illustrated in Equation (8) below:

(8) $$Purity=\frac{Number\;of\;Target\;Particles\;in\;the\;Selected\;Outlet}{Total\;Number\;of\;All\;Particles\;in\;the\;Selcted\;Outlets}\times 100$$

Particles’ sorting efficiencies and purities are reported as the average and the standard deviation of three measurements for three different tests (i.e., a total of 9 measurements).

## 3. Results and Discussion

Equations (1)–(3) can enable accurate estimation of the inertial and Dean drag forces acting on microparticles and the fluid recirculation velocity in curved channels. They have been used to design particle washing devices, where target particles are separated and transferred to a clean buffer [49,50]. We previously implemented these correlations to design a rectangular microchannel (300 µm × 60 µm) with a radius of curvature *R* = 1.185 cm. Our channel could separate target 11 µm particles from smaller 4 µm particles and transfer them into a clean buffer at the end of the channel with an efficiency of ~86 ± 2% at a low total flow rate of *Q_t_* = 2.0 mL/min [50]. Here, a similar channel cross section is used to investigate the particle separation and washing processes at higher flow rates when multiple curves are parallelized on one device as explained in the methods section.

Particle equilibrium positions in curved microchannels can be determined using the balance between the net inertial lift (*F_L_* in Equation (1)) and the Dean drag (*F_D_* in Equation (2)) forces. This ratio (*R_f_* = *F_L_*/*F_D_*) depends on particle size and flow conditions. In curved microchannels, under the dominant effect of inertial forces (*R_f_* ≫ 1), the particles tend to migrate towards the inner channel wall close to the center of curvature. On the other hand, when the Dean drag is dominant (i.e., *R_f_* ≪ 1), the Dean drag will entrain the particles towards the channel outer wall. Manipulation of the balance between net inertial lift forces and the Dean drag could lead to a sized-based particle separation in curved microchannels, while controlling the Dean fluid recirculation can provide particle washing simultaneously.

### 3.1. Fluid Switching in 2-Curve Channel Devices Featuring Rectangular and Polar Arrays

**Figure 5:**
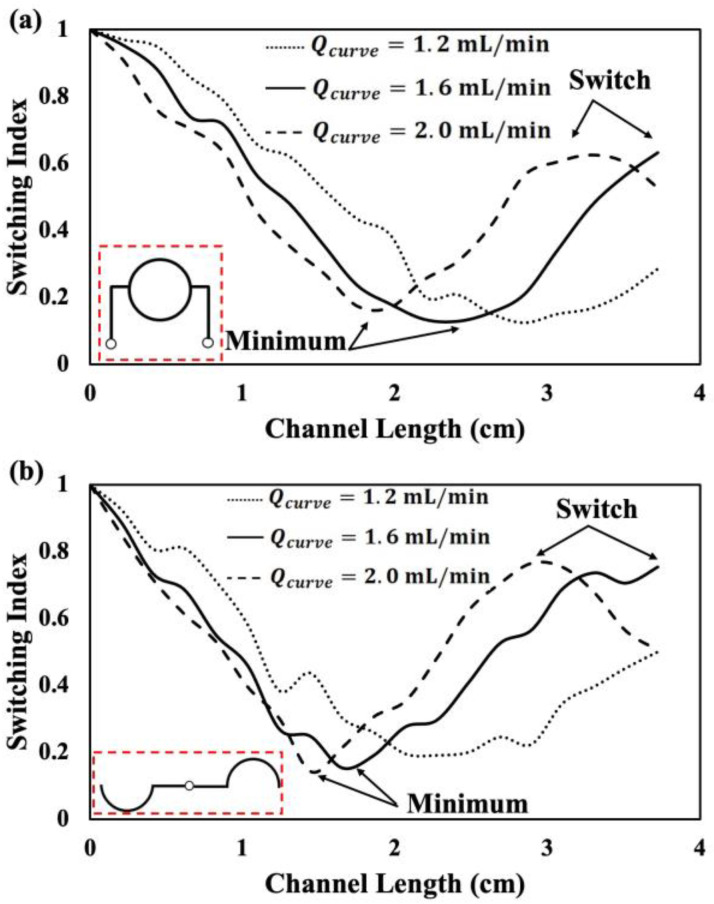
The switching index of dyed and undyed DI water flowing through one of the curve channels of (**a**) rectangular and (**b**) polar devices with 2 curves at three different flow rates. The first local maximum of *SI* indicates the fluid switching point due to the Dean flow recirculation. Red dotted boxes show the schematic of the used 2-curve microfluidic devices.

Initially, we investigated whether parallelization of the curved channel from one [50,56] to two curves, in rectangular and polar arrays, would impact the quality of fluid recirculation and switching. Figure 5 illustrates the effect of flow rate on the switching index (i.e., fluid recirculation) alongside the channel length in one of the curves, when the co-flows are supplied through the 2-curved devices with rectangular (Figure 5a) and polar (Figure 5b) configurations. 

As shown in Figure 5a, when the co-flows are supplied with a total flow rate of *Q_t_* = 4.0 mL/min (i.e., *Q_curve_* = 2 mL/min in each curve), the first fluid switch occurred before the curved channel outlets at *L_s_* = 3.28 cm. However, when the total flow rate was decreased to *Q_t_* = 3.2 mL/min, the switching index reached the local maximum at the channel outlet (*L_s_* = 3.72 cm). Upon further decrease in the flow rate to *Q_t_* = 2.4 mL/min, fluid recirculation was delayed and fluid switch did not happen by the channel outlet. 

As shown in Figure 5b for the 2-curved device with polar configuration, at a total flow rate of *Q_t_* = 4.0 mL/min, fluid switch occurred before the channel outlet at *L_s_* = 2.9 cm. When the flow rate decreased to *Q_t_* = 3.2 mL/min, switching index reached to the local maximum of *SI* = 0.75 at the channel outlet, which indicated the 0.5 fluid recirculation. A further decrease in the total flow rate down to *Q_t_* = 2.4 mL/min, resulted in no fluid switch at the channel outlet. 

All in all, we concluded that (1) the rectangular and polar array configurations behaved the same in fluid switching in 2-curve devices, and (2) despite the theoretical optimum flow rate of *Q_t_* = 4.0 mL/min [50] needed for a 0.5 recirculation, the fluid switch occurs at the total flow rate of *Q_t_* = 3.2 mL/min. This total flow rate was chosen for the continuation of this study in the next sections. This deviation from the theoretical flow rate could be due to the possible deformation of flexible PDMS channel walls at higher flow rates and small deviations of channel dimensions in the fabrication process. Moreover, the empirical *V_De_* correlation (Equation (3)) does not capture the effects of some parameters such as the channel material, i.e., rigid glass at one side and flexible PDMS at all other sides, that needs to be investigated in the future.

### 3.2. Effect of Channel Parallelization Design on Solution Exchange Efficiency 

**Figure 6:**
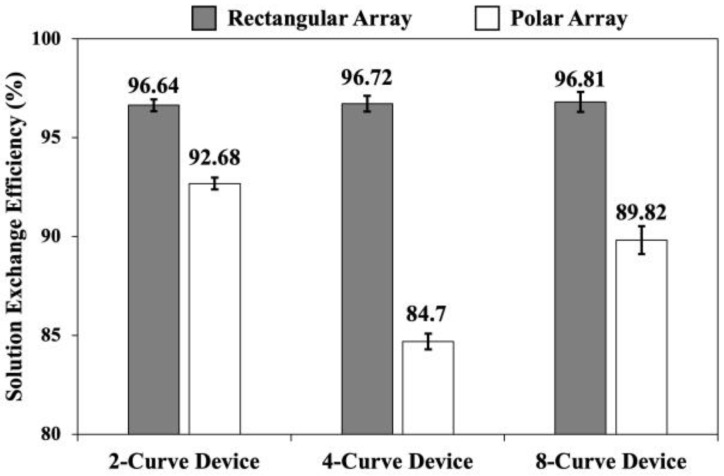
Solution exchange efficiency at the channel inner outlet for 2-curve, 4-curve, and 8-curve microdevices with rectangular and polar arrays, when the solutions are co-flown at *Q_curve_* = 1.6 mL/min in each curve (*Q_t_* = 3.2, 6.4, and 12.8 mL/min, respectively). The error bars indicate the standard deviation of three samples.

Solution exchange efficiencies at the devices inner outlets were investigated when the dyed and undyed solutions were co-flown at *Q_curve_* = 1.6 mL/min in each of the curves of the 2-curve, 4-curve, and 8-curve devices with rectangular and polar configurations (Figure 6). Here, the collected samples from the inner outlets were examined with the UV-VIS spectrophotometer, and the equivalent trypan blue concentrations in each sample were calculated using the calibration curve presented in Equation (6) and Figure 4b. 

As illustrated in Figure 6, for the co-flows in the 2-curve device with rectangular and polar arrays, solution exchange efficiencies were 96.64 ± 0.28% and 92.68 ± 0.32% respectively. Moreover, for the co-flows in the 4-curve device, the microdevice with the rectangular array resulted in a purity of 96.72 ± 0.37%. However, the solution exchange efficiency in the 4-curve device with a polar array dropped to 84.69 ± 0.40%. Finally, in the 8-curve device with the rectangular array, the collected sample at the inner outlets indicated a solution exchange efficiency of 96.81 ± 0.54%. On the other hand, the solution exchange efficiency in the 8-curve device with a polar array was 89.82 ± 0.67%. We concluded that, while the solution exchange efficiencies in the rectangular array remain fairly constant, it significantly decreases and fluctuates in the curve devices with a polar array. The poor performance of the polar device was because of the increased number of inlet and outlet ports compared to the rectangular device in which some inlets and outlets could be merged (Figure 2). This increase in the number of outlets led to unfavorable flow fluctuation inside the curves and lowered the solution exchange efficiencies in polar devices. Moreover, vicinity of the outlets to the outer edge of the device in the polar devices contributed to downstream changes in flow patterns due to the edging effects in photolithography and variation of edge thicknesses. In particular, for the 8-curve device with polar array the channel thickness varied between 64 µm to 76 µm, with higher channel thickness occurring in the vicinity of outer perimeter. In the rectangular devices, the inlets and outlets could be fit inside the curved channels and towards the center of the device (Figure 2c), therefore avoiding the effects of edge thickness variation (channel thickness variation was between 69 to 74 µm for the 8-curve device). Overall, based on the presented results in Figure 6, and due to a better performance consistency in rectangular devices, we decided to pursue particle separation investigations in the curved devices with a rectangular array.

### 3.3. Particle Separation Efficiency and Purity in Rectangularly Arrayed Devices

**Figure 7:**
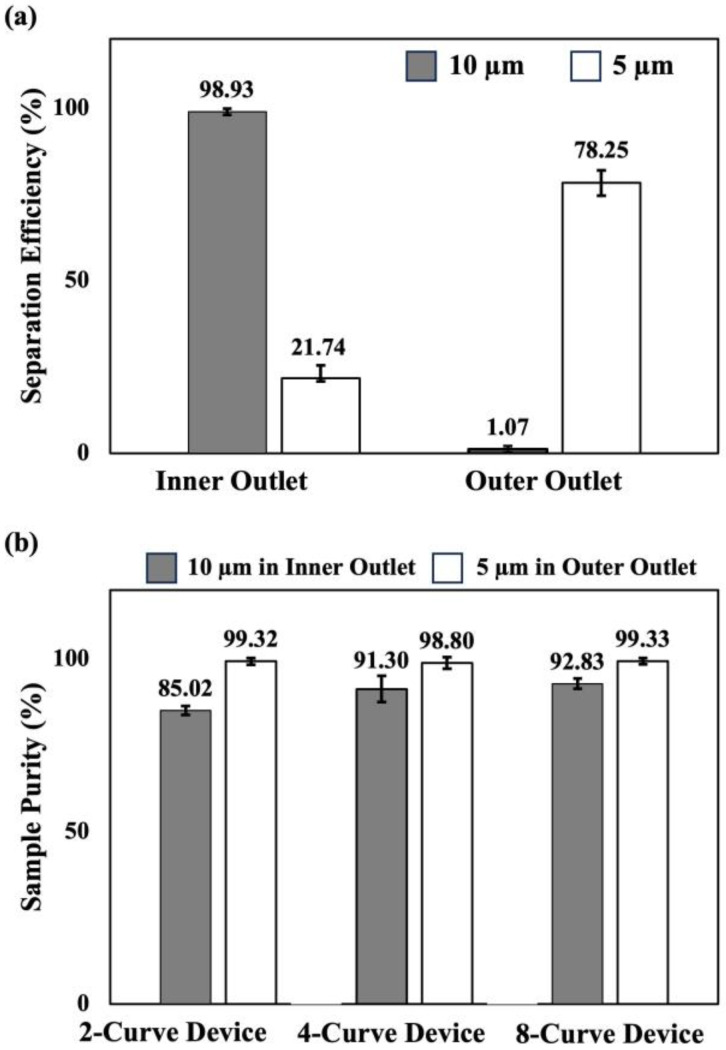
Separation efficiency and sample purity of the in-plane parallelized devices (**a**) Duplex separation efficiencies for 5 µm and 10 µm particles at the channel outlet, when the particle solutions were co-flown alongside a clean buffer at *Q_curve_* = 1.6 mL/min in the 2-curve device (*Q_t_* = 3.2 mL/min) with a rectangular array. (**b**) Sample purities for 5 µm and 10 µm particles in the inner and outer outlets, respectively, when co-flown simultaneously at *Q_curve_* = 1.6 mL/min in the 2-curve, 4-curve, and 8-curve microdevices with rectangular array. Error bars indicate the standard deviations of the measurements for three different samples.

Particle separation efficiencies were initially examined in the 2-curve microdevice with a rectangular array. Figure 7a shows the separation efficiencies when the duplex particle solution (mixture of 5 and 10 µm) is co-flown from the inner inlet alongside a clean buffer at a flow rate of *Q_t_* = 3.2 mL/min. Here, the larger 10 µm particles mostly remained close to the channel inner wall under the dominant effect of the inertial lift forces (*R_f_* = 4.7) and were collected with an average efficiency of 98.93 ± 0.91% in the inner outlet. On the other hand, the smaller 5 µm particles were under the dominant effect of the Dean drag (*R_f_* = 0.6) and were transferred towards the channel outer wall. As illustrated in Figure 7a, only 21.74 ± 3.72% of the 5 µm remained close to the channel inner wall. Meanwhile, 78.25 ± 3.72% of the smaller particles were collected from the outer outlets.

Duplex sample purities were examined inside the rectangular array microdevices with 2, 4, and 8 curves at total flow rates of *Q_t_* = 3.2, 6.4, and 12.8 mL/min, respectively. Figure 7b shows the sample purities for 10 µm and 5 µm particles collected from the inner and outer outlets, respectively. Here, the larger 10 µm particles were collected from the inner outlet with a purity of 85.02 ± 1.28% in the 2-curve device. The sample purities for the 10 µm particles increased in the 4-curve and 8-curve devices to 91.30 ± 3.75%, and 92.83% ± 1.47%, respectively. This indicates that a lower number of smaller 5 µm particles remained close to the channel inner wall in the 4-, and 8-curve devices. Although the solution exchange efficiencies were comparable in all devices with the rectangular array (Figure 6), smaller particles might have traveled more uniformly towards the outer wall which resulted in the increased sample purity in Figure 7b. 

We also investigated the sample purities for 5 µm particles at the outer outlets. As shown in Figure 7b, 5 µm particles were collected with an efficiency of 99.32 ± 0.97%, in the outer outlet of the 2-curve microdevice. Sample purities for the 5 µm particles in the 4-curve and 8-curve devices were similarly close with 98.80 ± 1.70%, and 99.33 ± 0.94%, respectively. Here, since the larger 10 µm particles were under the dominant effect of inertial lift forces, they were mainly collected at the inner outlets. Therefore, the Dean drag only managed to push the smaller 5 µm particles toward the outer wall. This resulted in high sample purities for smaller particles at the outer outlets.

Comparing the findings of our current investigation with those from our previous study [50], it is obvious that our new 8-curve parallelized device demonstrates a notable improvement. The parallelized device exhibits an enhanced capacity to retain 5 µm particles within the carrier fluid, achieving a purity level of 99.32 ± 0.97%. This shows an improvement over its previous value, which had a purity of 98 ± 0.7% [50]. Furthermore, our innovative device showed better performance for washing 10 µm particles and inertially focusing them at the inner outlet. This parallelization enhanced the sample purity at the inner outlet from 86 ± 2% in the prior device [50] to 92.83 ± 1.47% in our current configuration. Notably, this enhancement is achieved with an increase in flow rate by more than six folds. This difference could be due to more accurate fabrication process and a slight variation in particle sizes compared to the previous report. Moreover, the higher purities in this report are achieved after the optimization process while the prior report [50] mainly relied on the theoretical flow rate obtained from their empirical correlation [56]. In addition, it should be noted that each of our devices was utilized for an average of 4 h over a period of 10 days while devices were maintained inside a box at room temperature in the lab. This demonstrates the durability and continuous flow nature of our proposed technology. 

## 4. Conclusions

In summary, we investigated the effect of flow rate, and channel geometry on the performance of a particle washing process in a curvilinear microchannel system. Solution exchange and particle separation efficiencies were investigated with various flow rates in 2-, 4-, and 8-curve microchannels with rectangular and polar arrays. We found the optimum working condition and channel configuration and demonstrated a high throughput, high-efficiency particle separation and washing process. We achieved solution exchange efficiencies of >96.6% for high flow rates up to 12.8 mL/min. An efficient particle separation was demonstrated at a total flow rate of 12.8 mL/min for 5 µm, and 10 µm particles, where larger 10 µm particles were separated close to the channel inner wall with efficiencies > 98%. Sample purities of 92.83% and 99.33% were also achieved for 10 µm, and 5 µm particles in the inner and outer outlets, respectively. We aim to further enhance the throughput of the device via out-of-plane parallelization, improve its particle separation efficiency, and utilize this method to develop portable particle separation and washing technologies for biological samples. Pending possible modifications in channel dimensions based on the working fluid characteristics and sample size, this technology could enable separation and washing applications in blood diagnostics (i.e., red and white blood cells) and food and water monitoring (i.e., *E. coli* and microplastics).

## Data Availability

The data that support the findings on this study are available from the corresponding author upon reasonable request.
